# Glyphosate Determination by Coupling an Immuno-Magnetic Assay with Electrochemical Sensors

**DOI:** 10.3390/s18092965

**Published:** 2018-09-06

**Authors:** Francesca Bettazzi, Aline Romero Natale, Eduardo Torres, Ilaria Palchetti

**Affiliations:** 1 Dipartimento di Chimica, Università degli Studi di Firenze, Via della Lastruccia 3, 50019 Sesto Fiorentino (Fi), Italy; francesca.bettazzi@unifi.it; 2 Centro de Química-ICUAP, Benemérita Universidad Autónoma de Puebla, 72570 Puebla, Mexico; aline_natale@hotmail.com (A.R.N.); eduardo.torres@correo.buap.mx (E.T.)

**Keywords:** glyphosate, magnetic beads, electrochemical assay, disposable electrodes, beer

## Abstract

Glyphosate (*N*-(phosphonomethyl)glycine) is the most frequently used broad-spectrum herbicide worldwide. Its mechanism of action is based on the inhibition of an enzyme that is essential to plant growth. Its intensive use has caused global contamination to occur, which has not only affected the ecosystems, but even food and other objects of common use. Thus, there is a pronounced need for developing analytical methods for glyphosate determination in different matrices. Here, an electrochemical competitive immunoassay, based on the use of antibody-modified magnetic particles, has been developed. Tetramethylbenzidine (TMB) has been used as an enzymatic substrate. The extent of the affinity reaction has been achieved by monitoring the current value, due to the reduction of the enzymatic product. A disposable screen-printed electrochemical cell has been used. The calibration curve has been recorded in the 0–10,000 ng/L concentration range, with a detection limit of 5 ng/L and quantification limit of 30 ng/L. The electrochemical immunoassay has also been applied to the analysis of spiked beer samples.

## 1. Introduction

Glyphosate (*N*-(phosphonomethyl)glycine) is the most frequently used broad spectrum herbicide worldwide [1]. Glyphosate inhibits an enzyme that is essential to plant growth and which is involved in the shikimate cycle for the production of aromatic amino acids. This enzyme is also present in some bacteria, algae, and fungi, but not in mammals, birds, or fish.

Glyphosate is used primarily to combat weeds and, to a lesser extent, as pre-harvest treatment. Total glyphosate use worldwide (both agricultural and non-agricultural) has increased more than 12-fold, from about 67 million kg in 1995 to 826 million kg in 2014 [2]. Over the last decade, 6.1 billion kg of glyphosate have been applied, which is 71.6% of total use worldwide from 1974–2014 [2]. Since its introduction to the market in the early 70’s, glyphosate has been the subject of regular assessments by national and international regulatory agencies which established that glyphosate had relatively low toxicity in mammals [3]. However, the tremendous increase in its use has raised concerns about its ecotoxicological impacts and human risk, and there is also ongoing scientific and societal debate regarding the potential carcinogenicity of this herbicide [3]. In March 2015, the International Agency for Research on Cancer (IARC) classified glyphosate as being possibly carcinogenic to humans (Group 2A–IARC Monographs Programme) [4], and recently, the Californian Environmental Protection Agency decided in July 2017 to label glyphosate as a carcinogen. However, the IARC’s conclusion was not confirmed by other agencies [3], and the European Commission decided in December 2017 to relicense glyphosate for another 5 years. However, in September 2017, the European Commission made available to the Member States, the European Food Safety Authority’s (EFSA) conclusion on the potential endocrine-disrupting properties of glyphosate. In European Union (EU), the maximum residue limits (MRL) for different foodstuffs are listed in the pesticide database available on-line [5,6].

Indeed, it is the case that a global contamination has occurred due to the intensive use of glyphosate, which has not only affected the soil and surface and underground water, but even food and other objects of common use [7]. For this reason, monitoring of glyphosate in environmental and food samples seems mandatory nowadays, as well as the development of rapid screening analytical tools.

Various methods for glyphosate analysis have been developed, most of them based on liquid chromatography (LC), gas chromatography (GC), or capillary electrophoresis (CE) analysis. Recently, cell-based sensors have been applied to the assessment of glyphosate’s toxicity [8], and commercially available colorimetric enzyme-linked immunosorbent assay (ELISA) kits have been used for glyphosate determination in different matrices [9,10,11]. Some of these methods require derivatization of the analyte [12], and due to its high polarity, it is difficult to extract from samples and to retain it on chromatographic phases. Thus, derivatization with fluorenylmethyloxycarbonyl chloride (FMOC-Cl) is a common procedure used to improve extraction and separation. Derivatization techniques can also be useful in introducing chromophore or fluorophore to the glyphosate molecule for determination by UV and fluorescence techniques, respectively, as well as for LC or CE analysis or to increase the volatility in GC. However, the derivatization procedure is not highly regarded by analysts, as it requires the optimization of a number of parameters (temperature, reaction time, concentration and purity of the reagents, laboratory handling time). For these reasons, direct analysis of glyphosate by LC/MS/MS instruments has been also reported [13]. The derivatization procedure for the measurement of glyphosate using immunoreagents, such as in ELISA tests, is due to the immunogenic properties of this low molecular weight compound. It is well-known that small molecules are not immunogenic by itself and thus have to be conjugated with a larger molecular weight molecule (such as a protein) to elicit an immune response and to obtain antibodies. A possible way to increase the affinity of an antibody for a low molecular weight compound is to convert the target analyte in the sample into a derivative that mimics the immunogen, through the chemical addition of a reagent that is identical or similar to the spacer or chemical linker. This derivatization of analytes has been shown to increase the sensitivity of the immunoassay. A common reagent used for derivatization is acetic anhydride [10].

As discussed in a recent review paper by Gauglitz et al. [14], despite the need for a massive monitoring programme, sensors and biosensors for fast and cheap glyphosate screening analysis, directly in the field, are still rare. The use of screening methods can permit the confirmatory method to be used only for positive findings, thus decreasing the number of samples to be analyzed and reducing the cost of the analysis. Furthermore, sensor- and biosensor-based assays could be useful for detecting glyphosate for both laboratory and field analysis. An optical immunosensor was reported by Gonzales-Martinez [15], where the immunosensor carries out on-line analyte derivatization prior to the assay and uses a selective anti-glyphosate serum, a glyphosate peroxidase enzyme tracer, and fluorescent detection. Under optimal conditions, the detection limit achieved was 0.021 μg/L. A colorimetric immunoassay, capable of determining glyphosate in the range of 0.01–100 mg/L, was developed by using gold nanoparticles as the label [16]. The same assay scheme was proposed in another study by using fluorescence magnetic nanoparticles, consisting of Co–B(core)/SiO_2_ (shell)/dye NPs [17]; limits of detection in the range of mg/L were reported.

To the best of our knowledge, no reports have yet been published on the development of an electrochemical immuno-assay for glyphosate determination. Indeed, in the literature, molecular imprinted polymer-based electrochemical sensors [18,19,20,21,22] have been reported for glyphosate determination but not antibody-based sensors.

Thus, we have optimized a competitive immunoassay procedure based on anti-glyphosate-antibody-modified magnetic beads. Immunomagnetic assays are claimed to be particularly useful for improving the analytical performance of electrochemical immunoassays, since the immunoreaction is performed on a different surface, with respect to electrochemical detection [23,24]. Competition has been obtained using a Horseradish Peroxidase (HRP) conjugated tracer. Oxidation of 3,3′,5,5′-tetramethylbenzidine (TMB) substrate by HRP in the presence of H_2_O_2_ generates an electroactive product, which is stable in acid solutions and can be easily determined by chronoamperometry. Glyphosate has been found in surface water [9], cereals, dairy, and other foods [14], and recently, traces of glyphosate have been also found in beer [25]. Thus, the optimized chronoamperometric immuno-assay has herein been used to analyze spiked beer samples. The sample pre-analytical treatment was reduced to a minimum and portable electrochemical instrumentation was used throughout the experiment, demonstrating the operability of the assay under in-field analysis conditions.

## 2. Materials and Methods

### 2.1. Reagents

Anti-glyphosate-IgG modified magnetic beads (MBs) and HRP-conjugated-glyphosate (tracer) were obtained from ABRAXIS LLC (Warminster, PA, USA). The diluent solution (distilled water with preservative and stabilizers), washing solution (preserved deionized water), substrate solution (ready-to-use mixture of TMB and H_2_O_2_ in an organic medium, pH 6; concentrations not specified by the provider), and the Glyphosate HS Derivatization Kit were provided with the Glyphosate HS ELISA Kit by ABRAXIS LLC.

Glyphosate (certified reference material (CRM), EPA 547 glyphosate solution), 1 g/L in deionized water, chromatographic purity 99.9%, was obtained from Supelco (Milan, Italy). The CRM solution was stored at 4 °C until use. Solutions at different concentrations were freshly prepared by serial dilution from the CRM solution, stored at 4 °C, and used within the same day of preparation.

Peroxidase from horseradish (HRP) was purchased from Sigma-Aldrich-Fluka (Milan, Italy). Potassium chloride, potassium dihydrogenphosphate, and other chemicals were acquired from Merck (Milan, Italy).

Water from the Milli-Q Water Purification System (Milipore, Feltham, UK), was used for all solution buffers.

### 2.2. Buffer Solution and Derivatization Reagent

Assay Buffer (Dissolved buffer salts, pH = 8), provided with the Glyphosate HS ELISA Kit, was obtained from ABRAXIS LLC.

Citrate/phosphate buffer pH = 5 (0.1 M) was prepared by using disodium hydrogen phosphate dihydrate and trisodium citrate dihydrate (Merck, Milan, Italy). All chemicals used in this study were analytical reagent-grade chemicals.

A derivatization reagent, provided with the Glyphosate HS ELISA Kit (ABRAXIS LLC), was used to modify glyphosate in the standard solutions as well as in the spiked samples. Specific information regarding the derivatization reaction is not available. Following the instructions provided by the manufacturer, the derivatization reagent was diluted with an appropriate volume of derivatization reagent diluent (ABRAXIS LLC) immediately before use.

### 2.3. Screen Printed Electrochemical Cell (SPEC)

Carbon screen-printed electrochemical cells (SPECs) were provided by Ecobioservices and Researches (EBSR), Firenze, Italy. Each strip consisted of a carbon-based working electrode (ø 3 mm), an Ag/AgCl pseudo-reference electrode, and a graphite-based counter electrode. Measurements were performed by dropping 100 μL of the measurement solution onto the planar electrochemical cell [26,27]. SPECs were used without any previous cleaning treatments or modification.

### 2.4. Electrochemical Measurement

Electrochemical measurements were performed using Palmsens potentiostat with its proper software package (Palmsens, Houten, The Netherlands). All electrochemical experiments were carried out at room temperature. Each potential is referred to the Ag/AgCl pseudo-reference electrode.

Characterization of the electrochemical response of the TMB solution was performed by cyclic voltammetry (CV), scanning the potential in the range from −0.2 to +0.8 V, step potential 2 mV, with a scan rate of 25 mV/s.

Chronoamperometric detection of the enzymatically generated TMB in its oxidized form was performed by applying a fixed potential in the range of −0.1 to +0.1 V. To this end, 100 µL of TMB ready-to-use enzymatic substrate was incubated with and without the addition of 1 µL of a solution containing HRP (in the range from 2.3 × 10^−11^ M to 2.3 × 10^−15^ M), dissolved in citrate/phosphate buffer. After 15 min the solution was transferred onto the SPEC, and the potential was applied—current values were collected for 60 s, with a sampling rate of 0.5 s. Unless otherwise mentioned, 20 µL of 2 M aqueous solution of H_2_SO_4_ was added before measurements were taken in order to stop the enzymatic activity of HRP. This resulted in a pH shift to acidic values and a complete conversion of the blue product to its yellow diimine form, which could then be quantified by chronoamperometric measurement.

### 2.5. Immunoassay Procedure

20 µL of glyphosate-standard, control (diluent/zero standard solution), or beer sample solution, respectively, was pipetted into a 1.5 mL vial. In the same vial, 80 µL of assay buffer was added and the 100 µL solution was vortexed. Then, 8 µL of the diluted derivatization reagent was added and each vial vortexed immediately. Then, the derivatization reaction was allowed to proceed for 10 min. Meanwhile, the stock anti-glyphosate-IgG modified MBs suspension was mixed thoroughly. After 10 min, 200 µL of this stock MB suspension was added to each vial and briefly vortexed. After 30 min, 100 µL of glyphosate-enzyme-conjugate was added. Mixtures were vortexed and incubated for 30 min at room temperature. Each vial was then placed onto the magnetic separation stand (Promega, Milan, Italy) for 2 min to collect MB on the inner vial wall. The solution was then completely removed, and 400 µL of washing solution was added in each vial. The washing procedure was repeated twice with 200 µL and 100 µL of the washing solution, respectively, after which the vials were removed from the magnetic stand. 60 µL of the substrate solution was then added to each vial and, after 2 s of mixing, was allowed to incubate for 20 min. Finally, MBs were magnetically removed and the solution was completely transferred onto the electrochemical cell for chronoamperometric measurement.

### 2.6. Calibration Plot

The calibration plot for the standard solution of glyphosate was fitted by non-linear regression to the four-parameter logistic (4-PL) equation using Origin Pro 2018 software (Origin Lab Corporation, Northampton, MA, USA) according to the following formula:

$$\;y=A_{2}+\frac{\left(A_{1}-A_{2}\right)}{\left(1+{\left(\frac{x}{x_{0}}\right)}^{p}\right)}\;$$

where *y* is the analytical signal, A_2_ is the analytical signal at infinite analyte concentration, A_1_ is the analytical signal at zero analyte concentration, *x* is the analyte concentration, *x*_0_ is the inflection point on the calibration curve (the analyte concentration necessary to have 50% of the signal), and p is the slope parameter that defines the steepness of the curve [28,29,30]. The calibration curve was reported as %B/B_0_ versus the concentration of standard. The %B/B_0_ value was calculated for each standard by dividing the mean current value for the glyphosate standard solution by the mean current value for the zero standard solution.

The limit of detection (LOD) value was evaluated considering the mean response of the zero standard solution (B_0_) plus three times the standard deviation, whereas the limit of quantification (LOQ) was estimated considering the average response plus 10 times the standard deviation. Thus, the percentage of response at the LOD relative to B_0_ is calculated as %B/B_0LOD_ = [B_0_ + (3 × SD)]/B_0_ and the percentage of response at the LOQ relative to B_0_ is calculated as %B/B_0LOQ_ = [B_0_ + (10 × SD)]/B_0_. The obtained values were converted into ng/L by fitting the data to the 4-PL equation. The LOD is the minimum concentration of an analyte that can be distinguished from the assay background at a specified level of confidence. The LOQ is the minimum concentration that can be quantified at a specified level of precision. Thus, LOQ sets the lower limit for quantifying the analyte [31].

The upper limit of quantification (ULOQ) was considered as the highest amount of an analyte in a sample that can be quantitatively determined based on a specific degree of confidence. For this application, the ULOQ was considered as the concentration giving %B/B_0_ = 20%; this value was then interpolated on the standard curve. The dynamic range was considered to be the range of concentration (including ULOQ and LOQ) that can be reliably and reproducibly quantified with accuracy and precision through the use of a concentration–response relationship.

### 2.7. Matrix Effect

The matrix effect percentage (ME%) was defined as |ME%| = |[(Slope_linear regression matrix_/Slope_linear regression standard_) × 100] − 100|. For this purpose, calibration curves performed on a chosen concentration range (linear range) were fitted using linear regression (least squares method).

Furthermore, an F-test was applied to compare the squared standard errors (s^2^_y_) of the two regressions in each solution (matrix and standard).

### 2.8. Real Samples

Beer samples were purchased at a local food store. The beer samples were diluted 1:4 (*v*/*v*) in the diluent solution. Spiked samples were prepared by adding known amounts of glyphosate to diluted beer samples. The spiked samples were analyzed by the optimized electrochemical immunoassay and by a commercial ELISA kit.

The calibration plot for spiked glyphosate beer samples was fitted by linear regression (least squares method) using Origin Pro 2018 software, version 95E.

## 3. Results and Discussion

The electrochemical immunoassay was developed following a competitive assay scheme, using HRP-conjugated glyphosate molecules as a tracer. Among the different substrates for HRP-based detection, 3,3′,5,5′-Tetramethylbenzidine (TMB) is one of the most commonly used, and nowadays, several formulations with enhanced stability and solubility in aqueous buffer solution are commercially available. In the presence of H_2_O_2_, HRP catalyzes the oxidation of TMB (an aromatic amine) to the corresponding diimine in a two-electron oxidation process, following the equation:

TMB_(red)_ + H_2_O_2_ ⟶ TMB_(ox)_ + 2 H_2_O

The enzymatic activity can be monitored by the chronoamperometric measurement of the reduction current generated by TMB_(ox)_ at an appropriate working electrode. Thus, preliminary experiments were devoted to characterize the enzymatic reaction at the carbon screen-printed electrode surface.

### 3.1. Electrochemical Characterization of the Enzymatic Reaction at the Carbon Screen-Printed Electrodes

**Figure 1:**
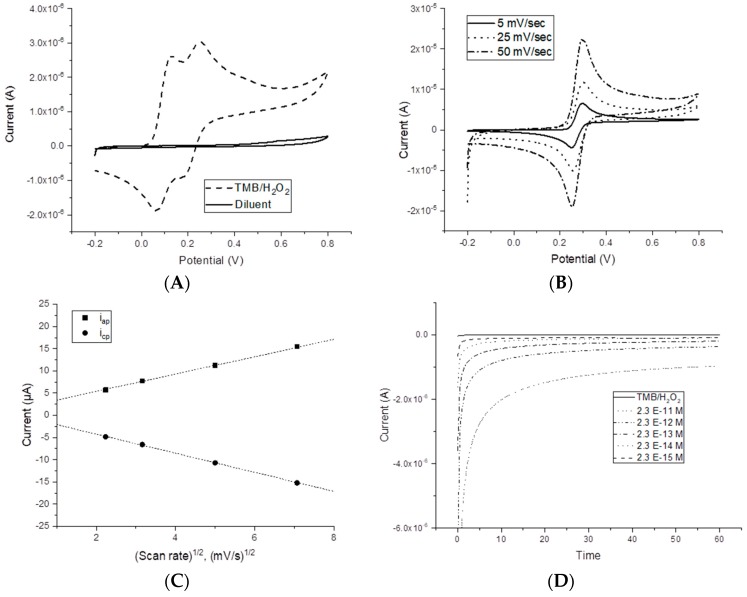
Electrochemical characterization: (**A**) Cyclic voltammogram for the Tetramethylbenzidine (TMB)/H_2_O_2_ solution recorded at the carbon screen-printed electrode (dash curve) in the −0.20 to +0.8 V potential range. The cyclic voltammetry (CV) for the diluent solution (solid line) is also reported. The potential was scanned with a scan rate of 25 mV/s; (**B**) cyclic voltammogram for the TMB/H_2_O_2_ solution recorded at the carbon screen-printed electrode in 0.5 M H_2_SO_4_ in the −0.20 to +0.80 V potential range, at different scan rates; (**C**) plot of i_p_ vs. square root of the scan rate, for a different scan rate; (**D**) chronoamperometric responses for different concentrations of HRP in 0.1 M citrate/phosphate buffer, pH 5.0, added to TMB/H_2_O_2_ solution recorded at the carbon screen-printed electrode with −0.1 V applied potential.

Figure 1A shows the Cyclic Voltammetry (CV) of a commercial, ready-to-use, TMB/H_2_O_2_ solution. Two oxidation peaks corresponding to the two-electron transfer reaction of TMB at potentials around +0.120 V and +0.286 V are present, while the two reduction peaks take place at around +0.047 V and +0.186 V. In H_2_SO_4_, the CV for TMB is characterized by a well-defined redox behavior: both cathodic and anodic peak potentials are independent of the scan rate (Figure 1B) with |E_p_-E_p/2_| = 30 ± 2 mV, thus around the expected Nernstian value for a two-electron reversible process [32,33]. Moreover, the i_p_ increases with the square root of the scan rate, demonstrating diffusion control of the current (Figure 1C).

Chronoamperometric hydrodynamic voltammetry for TMB_(ox)_ in the range −0.1 V to +0.1 V was carried out in order to choose the optimal working potential. A value of −0.1 V was chosen for the measurement of HRP enzymatic activity, as a good compromise between the faradic and background current. Indeed, substrate electrochemical oxidation should be avoided at this working potential, and the low value of the background current at −0.1 V is useful for determining small amounts of TMB_(ox)_ and thus of the enzyme molecules.

The chronoamperometric profiles of different HRP concentrations in 0.1 M citrate/phosphate buffer, pH 5.0, recorded at −0.1 V, in TMB/H_2_O_2_ solution are reported in Figure 1D. A linear relationship with the concentration of HRP was observed in the range of 2.3 × 10^−13^ to 2.3 × 10^−15^ M, sampling the current at 10 s. The corresponding equation was y = −1.4 × 10^6^x − 9.7 × 10^−8^. The LOD (calculated from the linear regression equation as the concentration corresponding to three times the standard deviation of the intercept) was 1.6 × 10^−15^ M. This value appears promising for using HRP as an enzymatic label coupled with the proposed electrochemical cell.

**Figure 2:**
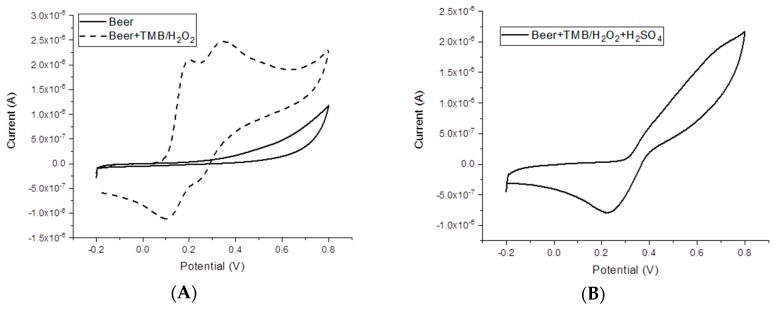
Electrochemical characterization of TMB at the screen-printed electrochemical cells (SPEC) in beer samples: (**A**) Cyclic voltammogram for the TMB/H_2_O_2_ solution added to a diluted beer sample recorded at the carbon screen-printed electrode (dash curve) in the −0.20 to +0.8 V potential range. The CV for the diluted beer sample (solid line) is also reported. The potential was scanned with a scan rate of 25 mV/s; (**B**) cyclic voltammogram for the TMB/H_2_O_2_ solution added to a diluted beer sample recorded at the carbon screen-printed electrode in presence of 0.5 M H_2_SO_4_ in the −0.20 to +0.8 V potential range. The potential was scanned with a scan rate of 25 mV/s.

Finally, the effect of the beer matrix composition on the electrochemical signal was evaluated. Figure 2 shows the CV of diluted beer samples before and after the addition of TMB/H_2_O_2_ solution. No redox signals are present in the CVs of the diluted beer samples, whereas after the addition of TMB/H_2_O_2_, the redox peaks of TMB are present. In the presence of 0.5 M H_2_SO_4_, the reduction peak potential at around +0.27 V is still well-defined, whereas a broad oxidation band could be observed, in comparison to the oxidation peaks observed in aqueous solution.

In order to check the effect of the matrix on the enzyme activity, the chronoamperometric profiles at different HRP concentrations in the diluted beer samples were recorded. A linear relationship with the concentration of HRP could still be observed, even if the current values were lower than that recorded in standard solution (data not shown).

These preliminary experiments confirm the possibility of directly performing the electrochemical measurement in beer samples. However, in order to avoid any possible adverse effect of sample components on the electrode surface and the reagents, the immunoassay was performed using magnetic beads as solid support for the affinity reaction. Many washing steps can be easily performed by using this strategy. Moreover, the electrode surface only serves for the chronoamperometric measurement because the affinity reaction and the incubation within the beer samples are performed onto the magnetic beads.

### 3.2. Optimization of the Electrochemical Immunoassay

**Scheme 1:**
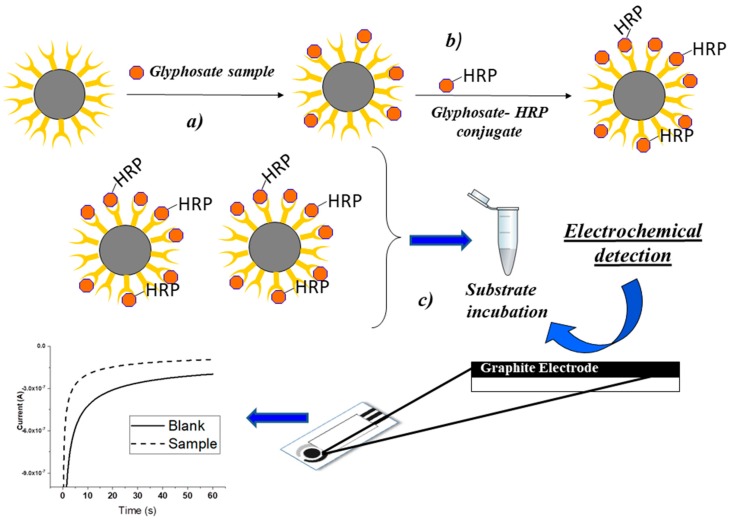
Scheme of the electrochemical immunoassay coupled with disposable screen-printed carbon electrode: (**a**) Paramagnetic beads modified with anti-glyphosate antibodies were incubated with samples; (**b**) a solution containing horseradish peroxidase (HRP) conjugate was added; (**c**) after several washing steps, the particles were magnetically blocked, and the substrate was added. The enzymatic product was measured by chronoamperometry.

The electrochemical immunoassay was developed using commercially available magnetic beads, modified with an anti-glyphosate antibody. A competition was then performed by incubating the corresponding tracer (HRP-conjugated glyphosate) at the optimized dilution in the presence of the sample. After the biomolecular recognition, the extent of the affinity reaction was evaluated by the addition of the enzymatic substrate TMB/H_2_O_2_. TMB was transformed in the oxidized form (TMB_ox_) and reduced at the electrode surface (Scheme 1).

Preliminary experiments were devoted to optimizing different parameters, and the results are reported in Table 1. Briefly, the optimal amount of MBs was found to be 200 µL, considering the best compromise between reagent consumption and the analytical signal. The addition of the optimal amount of MBs to the TMB solution presented no influence on the electrochemical measurements. Moreover, optimal TMB_(ox)_ detection was again performed at the potential equal to −0.1 V.

Afterwards, the inter-electrode reproducibility of the chronoamperometric signal and the current sampling time was evaluated. Using the zero-standard solution, the reduction current for n = 10 samples, after 10 s of applying the −0.1V working potential, was found to be −5.3 × 10^−7^ ± 0.7 × 10^−7^ A. After 20 s, the obtained current was −3.8 × 10^−7^ ± 0.1 × 10^−7^ A, whereas after 50 s, the value was −2.6 × 10^−7^ ± 0.1 × 10^−7^A. The standard deviation of the detected current after 20 s varied only by 3%, demonstrating that the electrodes can be used for immunoassays analysis. Moreover, this sampling time (20 s) was used for further measurements.

### 3.3. Analytical Evaluation of the Assay

**Figure 3:**
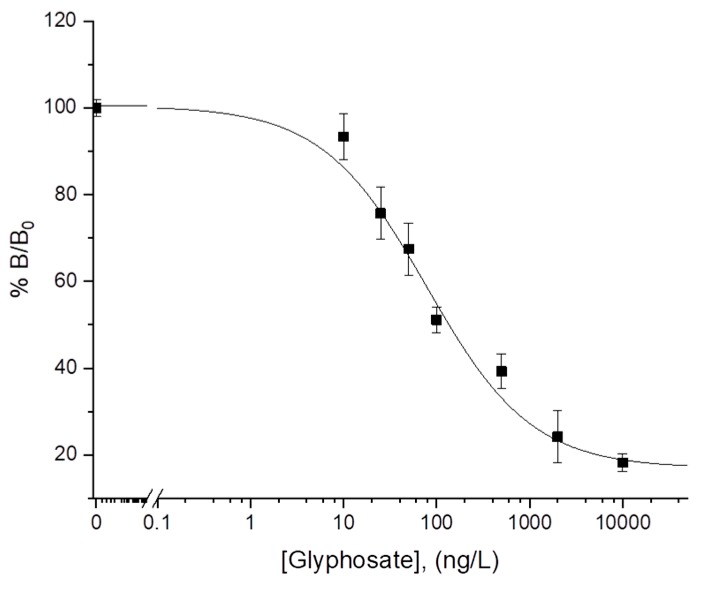
Glyphosate calibration curve. The experimental conditions are described in Table 1. The data points and the error bars represent average and standard deviation of measurements from different repetitions on the same standard (*n* = 3).

A calibration experiment was designed to demonstrate the analytical performances of the electrochemical assay. The 4PL equation was used to fit the data (R^2^ > 0.99, χ^2^ = 1.37). The limit of detection was evaluated to be 5 ng/L (2.96 × 10^−11^ M). The response of the assay spanned over three orders of magnitude, with a limit of quantification (defined as the background signal plus ten times its standard deviation) of 30 ng/L (1.77 × 10^−10^ M). The upper limit of quantification was 5400 ng/L (3.2 × 10^−8^ M). A typical calibration curve is shown in Figure 3. A dynamic range (concentration range between LLOQ and ULOQ) of 30–5400 ng/L was obtained, and the average RSD% was 8% (*n* = 3). Considering the reproducibility of the assay, the analytical signal that generates a normalized signal of 90% is considered as a cut-off value to delineate positive results from the negative. The analytical performances of the electrochemical assay were also evaluated in comparison with results obtained using the commercial ELISA colorimetric kit. The declared estimated minimum detectable concentration of colorimetric assay, based on a 90% B/B_0_, is 50 ng/L. Thus, the electrochemical assay seems to possess better performance in the lower end of the concentration range. Moreover, the LOD and the LOQ values of the proposed electrochemical immunoassay have been found to be lower than other LOD values recently reported for glyphosate detection by using sensors and biosensors (see Table S1 of Supporting Information) and the dynamic range comparable with the values reported for other methods, showing great potential for low cost, and preliminary screening application.

### 3.4. Application: Analysis in Beer Samples

**Figure 4:**
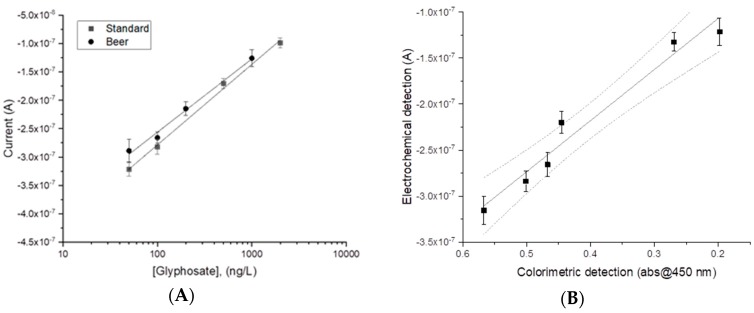
Measurements carried out in beer samples. (**A**) Calibration curve obtained with the chronoamperometric assay in commercial beer samples (circles). A calibration plot obtained in standard solution (squares) is also reported. The data points and error bars represent the average and standard deviation of measurements from three different repetitions on the same standard/sample (*n* = 3), and cathodic negative current values are reported; (**B**) correlation among the data obtained with the electrochemical immunoassay and a commercial colorimetric enzyme-linked immunosorbent assay (ELISA) on the same spiked beer samples (amount of glyphosate: 50, 100, 200, 500, 1000 ng/L). The data points and error bars represent the average and standard deviation of measurements from three different repetitions on the same standard/sample (*n* = 6).

Once the suitability of the assay to electrochemically detect glyphosate in standard solutions was verified, experiments on spiked commercial beer samples were carried out. The beer sample, diluted 1:4, was either tested alone or spiked with glyphosate. As expected, the current signal decreased with the increased concentration of the analyte, as shown in Figure 4A (curve beer).

The matrix effect was evaluated by comparing the slope of the linear regression obtained for the diluted beer samples with the slope obtained for standard solutions. In both cases, similar electroanalytical behavior, with a linear response toward the logarithm of glyphosate concentration, was obtained. The linear fitting for the analysis performed in standard solution was:

Current (A) = 1.41 × 10^−7^·log [glyphosate, ng/L] − 5.6 × 10^−7^, (r^2^ = 0.996)

On the other hand, the linear regression obtained for diluted beer samples was:

Current (A) = 1.21 × 10^−7^·log [glyphosate, ng/L] − 5.1 × 10^−7^, (r^2^ = 0.991). (1)

The F-test was applied to compare the squared standard errors (s^2^_y_) of the two regressions in each solution (standard and matrix) [34]. The experimental F value was clearly lower than the tabulated value (F_exp_ = 1.2, *n* (degree of freedom)_standard_ = 3, *n* (degree of freedom)_beer_ = 3) at a significance level of 5%, and so the null hypothesis could not be rejected (i.e., the variances of the two fits, calibration in matrix and calibration in standard, were not different). Nevertheless, the matrix effect percentage (ME%) was also evaluated and found to be 15%. Indeed, even if a possible interference from the matrix on the binding properties of the antibodies/modified magnetic beads on the enzymatic activity of the tracer and on the electrode fouling may not be excluded, a |ME% | ≤ 20%, is considered by several authors to have a negligible influence on analytical performance [35].

The same spiked beer samples were also analyzed by the commercial colorimetric ELISA kit obtaining a significant correlation at the level of 0.05 with a Pearson’s coefficient of r = 0.982 (degree of freedom *n* = 4). Thus, a linear correlation between the two techniques was found, shown in Figure 4B.

## 4. Conclusions

In this report, an electrochemical assay based on anti-glyphosate-antibody-modified MBs and disposable electrochemical sensors was developed for the determination of glyphosate in beer samples. The assay is cost-effective, since it involves a lesser amount of reagents in comparison to a colorimetric ELISA and low-cost, mass-produced sensors.

The electrochemical immunoassay showed a detection limit value in standard solution which was lower than the ones previously reported for other optical and electrochemical biosensors.

The LOD and LOQ for standard solutions were evaluated to be 5 ng/L and 30 ng/L, respectively, and the RSD% was 8%. The calibration curve carried out in beer samples covered a useful concentration range. The matrix effect was found to be acceptable (ME% = 15%), and results obtained with the proposed electrochemical assay correlated with results obtained using the colorimetric kit. The proposed strategy could serve as a basis for the development of rapid screening procedures for other food matrices.

## Figures and Tables

**Table 1:** Optimization of the different experimental parameters involved in the preparation of the electrochemical immunoassay, using anti-glyphosate-IgG magnetic beads (MB).

Parameter	Tested Range	Selected Value
Applied potential (V)	−0.1 ÷ 0.1 V	−0.1 V
Amount of MBs	500 ÷ 100 µL	200 µL
Amount of glyphosate-HRP	250 ÷ 100 µL	100 µL
Addition of H_2_SO_4_ after substrate incubation	yes/no	no

## Supplementary Materials

The following are available online at http://www.mdpi.com/1424-8220/18/9/2965/s1.
