# Supplementary material for: Glyphosate Determination by Coupling an Immuno-Magnetic Assay with Electrochemical Sensors
Source: Sensors (Basel). 2018 Sep 6;18(9):2965. doi: 10.3390/s18092965 (PMC6164882; doi:10.3390/s18092965)
Supplement: Supplementary file 1 [file sensors-18-02965-s001.pdf]

Glyphosate determination by coupling an immuno-magnetic assay with electrochemical sensors

Francesca Bettazzi[1], Aline Romero Natale[2], Eduardo Torres[2], Ilaria Palchetti[1]

[1]Dipartimento di Chimica, Università degli Studi di Firenze, Via della Lastruccia 3, 50019 Sesto Fiorentino (Fi), Italy

[2]Centro de Química-ICUAP, Benemérita Universidad Autónoma de Puebla, Puebla, Mexico

Corresponding author: ilaria.palchetti@unifi.it

Table 1: Comparison of the features of some sensors and biosensors recently reported in literature for glyphosate detection.

(Bio)sensing reagents	Assay type	Detection technique	Derivatization	LOD (as reported)	LOD (g/L)	Ref.
Antibody	Competitive	Fluorescence	yes	0.021 µg/L	21 ng/L	[1]
Oligopeptide	Direct	Surface plasmon resonance	no	0.58 µM	$9.8 \cdot 10^4$ ng/L	[2]
Antibody	Competitive	Fluorescence	no	8 ng/mL	$8 \cdot 10^3$ ng/L	[3]
Cu-dopped poly (vinyl) alcohol nanofibers + dithiocarbamic acid	Direct	Colorimetric	no	1.1 µg/mL	$1.1 \cdot 10^6$ ng/L	[4]
Ag ions	Binding-induced internal-displacement	Photoelectrochemical	no	30 pM	5.1 ng/L	[5]
Antibody	Sandwich	Fluorescence	no	0.01 µg/mL	$1 \cdot 10^5$ ng/L	[6]
Molecular imprinted polymer	Direct	Electrochemistry	no	0.35 ng/ mL	350 ng/L	[7]
Molecular	Direct	Electrochemistry	no	0.8 pg/L	$8 \cdot 10^{-4}$	[8]

imprinted polymer					ng/L	
Molecular imprinted polymer	Direct	Electrochemistry	no	1 pM	0.17 ng/L	[9]
Molecular imprinted polymer	Direct	Electrochemistry	no	0.27 ng /mL	270 ng/L	[10]
Antibody	Competitive	Electrochemistry	yes	5 ng/L	5 ng/L	This work

1. Gonzalez-Martinez, M. A.; Brun, E. M.; Puchades, R.; Maquieira, A.; Ramsey, K.; Rubio, F. Glyphosate Immunosensor. Application for Water and Soil Analysis. *Anal. Chem.* **2005**, *77*, 4219–4227.
2. Ding, X.; Yang, K.-L. Development of an Oligopeptide Functionalized Surface Plasmon Resonance Biosensor for Online Detection of Glyphosate. *Anal. Chem. (Washington, DC, United States)* **2013**, *85*, 5727–5733.
3.Wang, D.; Lin, B.; Cao, Y.; Guo, M.;Yu, Y. A Highly Selective and Sensitive Fluorescence Detection Method of Glyphosate Based on an Immune Reaction Strategy of Carbon Dot Labeled Antibody and Antigen Magnetic Beads *J. Agric. Food Chem*. **2016**, 64, 6042−6050
4. De Almeida, L.K.S.; Chigome S.; Torto N.; Frost C.L.; Pletschke B.I. A novel colorimetric sensor strip for the detection of glyphosate in water. *Sensors and Actuators B: Chemical* **2015**, 206, 357-363
5. Li, Y.; Zhang, S.; Zhang, Q.; Xu, G.; Dai, H.; Lin, Y. Binding-induced internal-displacement of signal-on photoelectrochemical response: A glyphosate detection platform based on graphitic carbon nitride. *Sensors Actuators, B Chem.* **2016**, *224*, 798–804.
6. Lee, H. U.; Shin, H. Y.; Lee, J. Y.; Song, Y. S.; Park, C. H.; Kim, S. W. Quantitative Detection of Glyphosate by Simultaneous Analysis of UV Spectroscopy and Fluorescence Using DNA-Labeled Gold Nanoparticles. *J. Agric. Food Chem.* **2010**, *58*, 12096–12100.
7. Prasad, B. B.; Jauhari, D.; Tiwari, M. P. Doubly imprinted polymer nanofilm-modified electrochemical sensor for ultra-trace simultaneous analysis of glyphosate and glufosinate. *Biosens. Bioelectron.* **2014**, *59*, 81–88.
8. Do, M. H.; Florea, A.; Farre, C.; Bonhomme, A.; Bessueille, F.; Vocanson, F.; Tran-Thi, N.-T.; Jaffrezic-Renault, N. Molecularly imprinted polymer-based electrochemical sensor for the sensitive detection of glyphosate herbicide. *Int. J. Environ. Anal. Chem.* **2015**, *95*, 1489–1501.
9. Mazouz, Z.; Kalfat, R.; Rahali, S.; Fourati, N.; Zerrouki, C.; Aloui, N.; Yaakoubi, N.; Seydou, M.; Chehimi, M. M.; Othmane, A. Highly Selective Polypyrrole MIP-Based Gravimetric and Electrochemical Sensors for Picomolar Detection of Glyphosate. *Sensors (Basel).* **2017**, *17*.
10. Zhang, C.; She, Y.; Li, T.; Zhao, F.; Jin, M.; Guo, Y.; Zheng, L.; Wang, S.; Jin, F.; Shao, H.; Liu, H.; Wang, J. A highly selective electrochemical sensor based on molecularly imprinted polypyrrole-modified gold electrode for the determination of glyphosate in cucumber and tap water. *Anal. Bioanal. Chem.* **2017**, *409*, 7133–7144.
